# Correction to “EHA Guidelines on management of chronic lymphocytic leukemia and Richter transformation”

**DOI:** 10.1002/hem3.70457

**Published:** 2026-08-07

Eichhorst B, Ghia P, Bosch F, et al. EHA Guidelines on management of chronic lymphocytic leukemia and Richter transformation. *HemaSphere*. 2026;10(6):e70403. doi:10.1002/hem3.70403.

In Figure 1, a level‐of‐evidence designation was omitted from one first‐line therapy treatment recommendation, and three typographical errors were identified. Figure 1 has been corrected accordingly.

Figure 2 was adapted from a previously published source. The appropriate attribution has now been added, and the corresponding reference has been included in the reference list. As a result, subsequent references and in‐text citations have been renumbered.

The original article has been updated. We apologize for these errors.

